# General Grant—Cancer of the Tongue

**Published:** 1885-05

**Authors:** 


### GENERAL GRANT—CANCER OF THE TONGUE

The intense interest with which the whole English-speaking people have watched the struggle with death, of the illustrious sufferer in New York, evidences the widespread sympathy which exists amongst them. In the presence of a great calamity, all past prejudices and antagonisms are forgotten. Actuated by a common sentiment of brotherhood, they mingle their tears and pour forth the tribute of their admiration for one of the noblest of the race. It is a touching historical picture.

The medical public is especially attracted to the terrible malady with which the great General is suffering. Cancer is not infrequent, but it seldom strikes so shining a mark. Regarded as incurable, the majority of its victims pass on unmarked to an inevitable destiny. But so celebrated a case cannot fail to renew inquiries in regard to it, and bring into review all that is known of its history, causes, diagnosis, pathology, medical and surgical treatment.

Cancer is almost universally regarded as a constitutional disease, dependent upon a certain crasis, which alone permits its development; but while its local origin is denied, it is admitted that various irritants, continuously applied, favor its development in particular locations. In the absence of these, it may make its appearance in any tissue of the body. Observation shows that it exhibits well-marked and constant preference for certain organs in different degrees. Nokitansky establishes the average scale of frequency of this preference as follows: First, the uterus, the female breast, the stomach, the rectum, the lymphatic glands, the bones, the skin, the lips, the brain, the eye, the testis, the ovary, the kidney, the tongue, the œrophagus, the salivary glands. If this classification be correct, cancer of the tongue ought to be rare, and the general observation of physicians we think justifies the conclusion. Cancer in other organs is of frequent occurrence; in tongue it is uncommon—not exceeding perhaps ten per cent. of the whole number.

If the cause of cancer is to be found in the degree or duration of local irritation to which an organ is exposed, the tongue should be its favorite seat. No organ of the body is subjected to such rough treatment and by so many irritants. In every case it is easy to find some local causes to which it is attributed—broken or decayed teeth, accumulations of tartar, bad dental work, false teeth, hot drinks, pepper pickles, or pungent spices, and more commonly, the use of alcohol and the smoking or chewing of tobacco.

Some of these accidents or habits are so nearly universal amongst mankind that the probability of any of them being the cause of the rare cases of cancer of the tongue is reduced to the minimum.

Hereditary or constitutional predisposition may doubtless result n cancer of the tongue in a proportion of frequency, determined by observation, but an estimate of its probability is an important aid to the diagnosis.

The tongue is liable to other affections, from which it is not easy to distinguish a cancer. It is claimed that by scraping the surface of a cancerous ulcer and placing the debris under a microscope the difference can always be made out. Frequent failures demonstrate that few, if any, possess the necessary skill. Scrofulous, syphilitic and indolent ulceration have been often mistaken for cancer, and turned over to the questionable risks of the surgeon's knife, when a more hopeful diagnosis would have mitigated suffering or brought a cure.

